# Changes in healthcare providers attitudes and practices about counseling pregnant and postpartum women about emergency preparedness –DocStyles survey, United States, spring 2021 and fall 2023

**DOI:** 10.1016/j.pmedr.2025.103261

**Published:** 2025-10-05

**Authors:** Jerome S. Leonard, Romeo R. Galang, Grayson Waits, Rebecca Hall, Carrie K. Shapiro-Mendoza, Jessica R. Meeker

**Affiliations:** a Division of Reproductive Health, Centers for Disease Control and Prevention, Atlanta, GA, United States; b Epidemic Intelligence Service, Centers for Disease Control and Prevention, Atlanta, GA, United States; c U.S. Public Health Service Commissioned Corps, Rockville, MD, United States

**Keywords:** Emergency preparedness, Pregnant women, Postpartum women, Preparedness counseling, Disasters, Maternal health

## Abstract

**Objective:**

We compared healthcare provider attitudes and practices related to counseling pregnant, postpartum, and lactating women (PPLW) about emergency preparedness in 2021 and 2023.

**Methods:**

A web-based survey of United States healthcare providers was administered in 2021 (*N* = 1503) and 2023 (N = 1503) by Porter Novelli DocStyles. We compared frequencies and 95 % confidence intervals (CIs) of provider attitudes and practices, and unadjusted prevalence ratios for questions with binary responses.

**Results:**

Compared to the 2021 sample, higher proportions of providers in the 2023 sample reported counseling patients on emergency preparedness as “Very Important” (31.0 % to 42.1 %) [95 % CIs: (28.7, 33.3); (39.6, 44.6)]; and reported to have counseled patients on emergency preparedness plans (29.8 % to 36.6 %) [95 % CIs: (27.5, 32.1); (34.2, 39.0)]. Providers reported similar levels of overall confidence counseling patients on emergency preparedness plans from 2021 to 2023 (53.2 % to 51.0 %) [(95 % CIs: (50.7, 55.7); (48.5, 53.6)]. However, OB/GYNs reported a significantly decreased level of overall confidence in such counseling (69.6 % to 52.4 %) [95 % CIs: (63.9, 75.3); (46.2, 58.5].

**Conclusions:**

These findings show healthcare providers increasingly recognize emergency preparedness counseling for PPLW as very important but there remains a need to build provider confidence in counseling PPLW on emergency preparedness.

## Highlights

• Providers increasingly report emergency preparedness counseling as very important.
• A majority of providers have never counseled women on emergency preparedness.
• Only 51 % of providers report feeling confident counseling on emergency preparedness.
• Obstetrician/gynecologists reported a 17.2 percentage point decrease in confidence.
• Obstetrician/gynecologists were the only specialty to report decreased confidence.

## 1 Introduction

Both natural disasters (e.g., hurricanes and wildfires) and man-made disasters (e.g., toxic exposures such as in Flint, Michigan or East Palestine, Ohio) create unique challenges for pregnant, postpartum, and lactating women (PPLW) (Harville et al., 2010; Zotti et al., 2013; Foo et al., 2024). Exposure to disasters is associated with adverse pregnancy outcomes including preterm birth and pregnancy loss (Harville et al., 2010; Mendez-Figueroa et al., 2019; Cordero, 1993). These adverse pregnancy outcomes can potentially be mitigated in part by having emergency preparedness plans in place (Ewing et al., 2008). Healthcare providers play critical roles in counseling PPLW on emergency preparedness planning, including mitigating physical and mental health risks during pregnancy and the postpartum period during and after disasters and severe weather emergencies.

While emergency preparedness plans can potentially mitigate some adverse pregnancy outcomes, we found in 2021 a minority (30 %) of providers reported having ever counseled women of reproductive age on this topic (Meeker et al., 2023). In the 2021 DocStyles survey, we also found that, while a majority of healthcare providers reported emergency plans were important (88 %) nearly half (45 %) of providers did not feel confident counseling their patients and most (70 %) had never provided emergency preparedness counseling with women of reproductive age (Meeker et al., 2023). Since the 2021 survey, the United States has faced over 200 federally declared disasters and public health emergencies (Disasters and Other Declarations, 2025), including Hurricane Ian – the third-costliest hurricane to strike the United States with an estimated $115 billion in damages – and the deadliest wildfires of the past century in Maui, with 100 fatalities (Billion-Dollar Weather and Climate Disasters, 2025). As the risk of disasters continue to increase in frequency and severity (Marvel et al., 2023), we compared survey data from 2021 (*N* = 1503) to 2023 (N = 1503) to assess recent changes in providers attitudes and practices in counseling on emergency preparedness for PPLW.

## 2 Methods

### 2.1 Participants

DocStyles is a web-based, non-probability panel survey of physicians and primary care providers practicing in the United States. Between October 6–October 25th, 2023, Porter Novelli commissioned M3 Global Research to conduct a DocStyles survey consisting of 500 family medicine physicians, 500 internal medicine physicians, 250 Obstetrician/Gynecologists (OB/GYNs), and 250 physician assistants and nurse practitioners providing primary care services in the United States. All U.S. States were represented in both 2021 (*N* = 1503) and 2023 (N = 1503) samples. The 2021 DocStyles survey administration is described in detail in Meeker et al. (Meeker et al., 2023). Sampling methodologies for both years are described at https://styles.porternovelli.com/docstyles. Respondents were paid an honorarium of $30–$50 for completing the survey based on the number of questions they were asked. DocStyles has previously been used to describe both provider attitudes (Meeker et al., 2023; Boothe-LaRoche et al., 2014) and changes to provider attitudes over time (Quader et al., 2017; Jones et al., 2021).

### 2.2 Data collection

For this study, we compared responses to four emergency preparedness questions specific to counseling needs of PPLW (Noted as EPR1-EPR4 in *Appendix 1*), which were asked in both 2021 and 2023 surveys. Respondents were asked about the importance of having emergency preparedness plans (EPR1), the importance of talking with PPLW about emergency preparedness (EPR2), if they had ever talked with PPLW about developing an emergency preparedness plan (EPR3) and level of provider confidence in talking with pregnant women (EPR4). Henceforth, “talking with PPLW” will be referred to as “counseling.” The overall response rate increased from 66 % in 2021 (65 % for internists and family practice providers, 62 % for OB/GYNs, and 71 % for nurse practitioners and physician assistants (NP/PAs) to 70 % in 2023 (72 % for internists and family medicine providers, 60 % for OB/GYNs, and 76 % for NP/PAs). The 2021 and 2023 survey respondents were compared for differences in self-reported characteristics, including provider types, sex, age, years of clinical practice, patients per week, practice type (*group outpatient, individual outpatient, inpatient*), community type (*rural, suburban, urban*), U.S. census region (*midwest, northeast, south, west*) and approximate financial situation (household income) of the majority of their patients (*Poor (Less than $25,000), Lower middle ($25,000 - $49,999), Middle ($50,000–$99,999), Upper middle ($100,000 - $249,999), Affluent ($250,000 or more*)). Terminology for EPR1 and EPR2 was changed to narrow the focus from “women of reproductive age (including pregnant, postpartum, or lactating women)” in 2021 to “pregnant, postpartum, or lactating women” in the 2023 survey. Answer options were expanded for specific questions on the 2023 survey, including adding “Don't Know” to level of provider confidence (EPR4). The 2021 and 2023 survey asked provider gender but has been changed to “sex” in this analysis to comply with U.S. government regulations. Sixteen respondents in 2023 were excluded from the analysis of sex as it was restricted to respondents who responded “Male” or “Female”, these respondents are included in the remaining analyses.

### 2.3 Statistical analysis

Differences in sample demographics between 2021 and 2023 were compared by Chi-Square testing for categorical variables (provider types, sex, age, years of clinical practice, practice type, community type, U.S. census region and patient income) and by *t*-test for continuous variables (patients per week). For all EPR questions, frequencies and 95 % confidence intervals (95 % CIs) were calculated for each response in both 2021 and 2023; differences between samples were considered significant if 95 % CIs did not overlap. For the 2023 sample, we performed bivariate analysis of whether counseling had ever been performed (EPR3) by provider characteristics and attitudes using unadjusted log-binomial models to estimate prevalence ratios of providers most likely to have reported counseling. Unadjusted bivariate analysis for the 2021 sample are described in Meeker et al. (Meeker et al., 2023) Similarly, prevalence ratios from unadjusted bivariate log-binomial models are presented for the 2023 data. Provider covariates were checked in multivariable analyses for confounding; however, effect sizes did not change by more than 2 %. As such, we present the unadjusted 2023 results only. All calculations were performed in R version 4.3.0 (R Foundation, Vienna, Austria).

This activity was reviewed by the Centers for Disease Control and Prevention (CDC) and was conducted consistent with applicable federal law and CDC policy. Data licensed from Porter Novelli do not include personal identifiers; the activity is considered non-human subjects research and analyses of DocStyles survey data do not require CDC Institutional Review Board approval.

## 3 Results

A total of 1503 respondents completed the survey in 2023, an equal amount to the 1503 respondents in the 2021 survey. No statistical differences were observed between 2021 and 2023 survey respondents by provider type, sex, number of patients per week, practice type, community type, census region or approximate patient income (Table 1). A statistical difference was observed in the distribution of provider age groups (*p* = 0.017) and years in practice (*p* < 0.001) between 2021 and 2023 samples. A majority of respondents in both samples were male (58 % and 54 %), worked in group outpatient practices (64 % and 67 %), practiced in suburban communities (53 % and 52 %). Respondents most commonly resided in the South (33 % and 37 %) and estimated a majority of their patients were “Middle Income ($50,000-$99,999)” earners (42 % and 44 %).

**Table 1:** Sample characteristics for total respondent groups -Fall DocStyles, US, 2023 and Spring DocStyles, US, 2021.

	2021 (*N* = 1503) N (%)	2023 (N = 1503) N (%)	P-Value^1^
Specialty			
Family Medicine	490 (32.6)	532 (35.4)	0.293
Internist	512 (34.1)	469 (31.2)	
NP/PA	251 (16.7)	250 (16.6)	
OB/GYN	250 (16.6)	252 (16.8)	
Age Group (in years)			
<35	237 (15.8)	269 (17.9)	0.017
35–50	660 (43.9)	590 (39.3)	
51–65	487 (32.4)	490 (32.6)	
>65	119 (7.9)	154 (10.2)	
Sex^2^			
Female	633 (42.1)	676 (45.0)	0.061
Male	870 (57.9)	811 (54.0)	
Patients Per Week			
Mean [SD]	100 [65.1]	102 [66.2]	0.549
Interquartile Range	50.0	60.0	
Years of practice			
3–5	198 (13.2)	268 (17.8)	<0.001
6–10	310 (20.6)	295 (19.6)	
11–20	484 (32.2)	382 (25.4)	
>20	511 (34.0)	558 (37.1)	
Practice type			
Group outpatient practice	959 (63.8)	1007 (67.0)	0.174
Individual outpatient practice	272 (18.1)	251 (16.7)	
Inpatient/Hospital	272 (18.1)	245 (16.3)	
Community			
Rural	162 (10.8)	171 (11.4)	0.855
Suburban	795 (52.9)	786 (52.3)	
Urban	546 (36.3)	546 (36.3)	
Census Region			
Midwest	330 (22.0)	323 (21.5)	0.218
Northeast	354 (23.6)	324 (21.6)	
South	497 (33.1)	549 (36.5)	
West	322 (21.4)	307 (20.4)	
Approximate patient household income			
Poor (Less than $25,000)	94 (6.3)	66 (4.4)	0.173
Lower middle ($25,000 - $49,999)	356 (23.7)	367 (24.4)	
Middle ($50,000–$99,999)	631 (42.0)	666 (44.3)	
Upper middle ($100,000 - $249,999)	288 (19.2)	281 (18.7)	
Affluent ($250,000 or more)	134 (8.9)	123 (8.2)	

NP – Nurse Practitioner; PA – Physician Assistant; OB/GYN – Obstetrician/Gynecologist; SD – Standard Deviation.

1 P-Values calculated by Chi-Square testing for categorical variables and by t-test for continuous variables.

2 16 respondents who selected “Prefer to self-identify” were excluded from sex (*N* = 1487) in 2023.

### 3.1 Importance of having emergency preparedness plans

Compared to 2021, a similar proportion of providers in 2023 reported that emergency preparedness plans were either “Somewhat” or “Very” important to have (2021: 87.6 % to 2023: 86.6 %). There was a significant decrease in providers reporting emergency preparedness plans as “Somewhat Important” (36.9 % to 31.0 %) [95 % CIs: (34.5, 39.4); (28.7, 33.3)] **(**Table 2**)**. There was a non-significant increase in providers reporting them as “Very Important” (50.7 % to 55.6 %) [95 % CIs: (48.2, 53.2); (53.1, 58.1)]. No significant changes over time were observed for reporting emergency preparedness plans as “Very Important” when stratified across all provider types.

**Table 2:** Percent of primary healthcare providers reporting various emergency preparedness and response counseling attitudes, by provider type - fall DocStyles, US, 2023 (N = 1503) and spring DocStyles, US, 2021 (N = 1503).

EPR 1: In general, how important are emergency preparedness plans for keeping pregnant, postpartum, or lactating women^1^ healthy in disasters or severe weather emergencies?										
	Overall		Family Medicine		Internist		Nurse Practitioner/Physician Assistant		Obstetrician/Gynecologist	
	2021 (*N* = 1503)	2023 (N = 1503)	2021 (*N* = 490)	2023 (*N* = 532)	2021 (*N* = 512)	2023 (*N* = 469)	2021 (*N* = 251)	2023 (*N* = 250)	2021 (N = 250)	2023 (*N* = 252)
*Response*										
Very Important	50.7 (48.2, 53.2)	55.6 (53.1, 58.1)	46.5 (42.1, 50.9)	54.1 (49.9, 58.4)	49.0 (44.7, 53.4)	52.5 (47.9, 57.0)	56.6 (50.4, 62.7)	60.4 (54.3, 66.5)	56.4 (50.0, 62.5)	59.9 (53.9, 66.0)
Somewhat Important	36.9 (34.5, 39.4)	31.0 (28.7, 33.3)	42.0 (37.7, 46.4)	33.8 (29.8, 37.9)	34.2 (30.1, 38.3)	32.6 (28.4, 36.9)	31.5 (25.7, 37.2)	24.0 (18.7, 29.3)	38.0 (32.0, 44.0)	29.0 (23.4, 34.6)
Not too Important	4.7 (3.7, 5.8)	4.7 (3.7, 5.8)	4.3 (2.5, 6.1)	4.3 (2.6, 6.1)	5.9 (3.8, 7.9)	4.9 (3.0, 6.9)	4.8 (2.1, 7.4)	4.8 (2.2, 7.4)	3.2 (1.0, 5.4)	5.2 (2.4, 7.9)
Not Important	1.1 (0.5, 1.6)	1.5 (0.9, 2.1)	1.2 (0.3, 2.2)	1.7 (0.6, 2.8)	1.2 (0.2, 2.1)	1.1 (0.1, 2.0)	0.4 (0, 1.2)	1.2 (0, 2.5)	1.2 (0, 2.5)	2.0 (0.3, 3.7)
Don't know	6.6 (5.3, 7.8)	7.2 (5.9, 8.5)	5.9 (3.8, 8.0)	6.0 (4.0, 8.0)	9.8 (7.2, 12.3)	9.0 (6.4, 11.5)	6.8 (3.7, 9.9)	9.6 (5.9, 13.3)	1.2 (0, 2.5)	4.0 (1.6, 6.4)
Overall Importance	87.6 (86.0, 89.3)	86.6 (84.9, 88.3)	88.6 (85.8, 91.4)	88.0 (85.2, 90.7)	83.2 (80.0, 86.4)	85.0 (81.8, 88.3)	88.0 (84.0, 92.1)	84.4 (79.9, 88.9)	94.4 (91.5, 97.3)	88.9 (85.0, 92.8)

EPR 2: How important is talking with^2^ pregnant, postpartum, or lactating women^1^ about emergency preparedness plans for disasters or severe weather emergencies?										
Very Important	31.0 (28.7, 33.3)	42.1 (39.6, 44.6)	26.3 (22.4, 30.2)	40.8 (36.6, 45.0)	33.6 (29.5, 37.7)	42.2 (37.8, 46.7)	36.7 (30.7, 42.6)	51.2 (45.0, 57.4)	29.2 (23.6, 34.8)	35.7 (29.8, 41.6)
Somewhat Important	45.5 (43.0, 48.0)	37.6 (35.1, 40.0)	49.0 (44.6, 53.4)	40.2 (36.1, 44.4)	41.0 (36.8, 45.3)	37.1 (32.7, 41.5)	43.0 (36.9, 49.2)	28.4 (22.8, 34.0)	50.4 (44.2, 56.6)	42.1 (36.0, 48.2)
Not too Important	12.8 (11.1, 14.5)	10.1 (8.6, 11.6)	13.7 (10.6, 16.7)	9.8 (7.3, 12.3)	12.1 (9.3, 14.9)	9.0 (6.4, 11.5)	9.6 (5.9, 13.2)	8.4 (5.0, 11.8)	15.6 (11.1, 20.1)	14.7 (10.3, 19.1)
Not Important	4.1 (3.1, 5.1)	2.9 (2.0, 3.7)	4.5 (2.7, 6.3)	3.2 (1.7, 4.7)	4.9 (3.0, 6.7)	1.7 (0.5, 2.9)	3.2 (1.0, 5.4)	2.0 (0.3, 3.7)	2.4 (0.5, 4.3)	5.2 (2.4, 7.9)
Don't know	6.7 (5.4, 7.9)	7.3 (6.0, 8.6)	6.5 (4.3, 8.7)	6.0 (4.0, 8.0)	8.4 (6.0, 10.8)	10.0 (7.3, 12.7)	7.6 (4.3, 10.8)	10.0 (6.3, 13.7)	2.4 (0.5, 4.3)	2.4 (0.5, 4.3)
Overall Importance	76.5 (74.4, 78.7)	79.7 (78.0, 81.7)	75.3 (71.5, 79.1)	81.0 (78.7, 84.3)	74.6 (70.8, 78.4)	79.3 (75.7, 83.0)	79.7 (74.7, 85.0)	79.6 (74.6, 85.0)	79.6 (74.6, 84.6)	77.8 (72.6, 82.9)

EPR 3: Have you ever talked with^2^ a pregnant, postpartum, or lactating woman about developing an emergency preparedness plan in case of a disaster or severe weather emergency?										
Yes	29.8 (27.5, 32.1)	36.6 (34.2, 39.0)	28.0 (24.0, 31.9)	36.5 (32.4, 40.6)	24.0 (20.3, 27.7)	30.5 (26.3, 34.7)	25.5 (20.1, 30.9)	41.6 (35.5, 47.7)	49.6 (43.4, 55.8)	43.3 (37.1, 49.4)
No	70.2 (67.9, 72.5)	63.4 (61.0, 65.8)	72.0 (68.1, 76.0)	63.5 (59.4, 67.6)	76.0 (72.3, 79.7)	69.5 (65.3, 73.7)	74.5 (69.1, 79.9)	58.4 (52.3, 64.5)	50.4 (44.2, 56.6)	56.7 (50.6, 62.9)

EPR 4: How confident are you in talking with^2^ a pregnant woman about developing an emergency preparedness plan (including preparing an emergency birth kit) in case of a disaster or severe weather emergency?										
Very Confident	15.0 (13.2, 16.8)	18.2 (16.3, 20.2)	16.3 (13.1, 19.6)	18.4 (15.1, 21.7)	11.7 (8.9, 14.5)	15.4 (12.1, 18.6)	14.3 (10.0, 18.7)	20.8 (15.8, 25.8)	20.0 (15.0, 25.0)	20.6 (15.6, 25.6)
Somewhat Confident	38.2 (35.7, 40.6)	32.8 (30.4, 35.2)	40.8 (36.5, 45.2)	33.6 (29.6, 37.7)	34.4 (30.3, 38.5)	30.3 (26.1, 34.4)	29.5 (23.8, 35.1)	36.8 (30.8, 42.8)	49.6 (43.4, 55.8)	31.7 (26.0, 37.5)
Not too Confident	32.2 (29.8, 34.6)	28.0 (25.7, 30.3)	31.8 (27.7, 36.0)	29.5 (25.6, 33.4)	35.7 (31.6, 39.9)	30.9 (26.7, 35.1)	32.7 (26.9, 38.5)	19.6 (14.7, 24.5)	25.2 (19.8, 30.6)	27.8 (22.2, 33.3)
Not Confident	14.6 (12.8, 16.4)	16.8 (14.9, 18.7)	11.0 (8.3, 13.8)	15.4 (12.3, 18.5)	18.2 (14.8, 21.5)	17.9 (14.4, 21.4)	23.5 (18.3, 28.8)	18.4 (13.6, 23.2)	5.2 (2.5, 8.0)	16.3 (11.7, 20.8)
Don't know^3^	N/A	4.1 (3.1, 5.1)	N/A	3.0 (1.6, 4.5)	N/A	5.5 (3.5, 7.6)	N/A	4.4 (1.9, 6.9)	N/A	3.6 (1.3, 5.9)
Overall Confidence	53.2 (50.7, 55.7)	51.0 (48.5, 53.6)	57.1 (52.8, 61.5)	52.0 (47.8, 56.3)	46.1 (41.8, 50.4)	45.6 (41.1, 50.1)	43.8 (37.7, 50.0)	57.6 (51.5, 63.7)	69.6 (63.9, 75.3)	52.4 (46.2, 58.5)

All numbers are % followed by (95 % CI).

Overall Importance/Confidence: Sum of Very and Somewhat Important/Confident

^1^The 2021 survey used the terminology “women of reproductive age (including pregnant, postpartum, or lactating women)”

^2^”Talking with” is referred to as “counseling” in the text

^3^“Don't Know” was added as an answer choice for the 2023 survey.

### 3.2 Importance of counseling on emergency preparedness plans

Overall, a similar proportion of providers in both years reported counseling PPLW about emergency preparedness plans as important (76.5 % to 79.7 %); however, an increased proportion reported this counseling as “Very Important” in 2023 (31.0 % to 42.1 %) [95 % CIs: (28.7, 33.3); (39.6, 44.6)] **(**Table 2**)**. A statistically significant increase was observed for all provider types, except OB/GYNs (non-significant increase of 6.5 percentage points). The largest increases in “Very Important” were reported among NP/PAs (36.7 % to 51.2 %) [95 % CIs: (30.7, 42.6); (45.0, 57.4)], and by family medicine providers (26.3 % to 40.8 %) [95 % CIs: (22.4, 30.2); (36.6, 45.0)].

### 3.3 Prevalence of counseling for emergency preparedness plans

In both years, a majority of providers had never counseled PPLW on emergency preparedness planning, although the proportion significantly decreased from 70.2 % in 2021 to 63.4 % in 2023 [95 % CIs: (67.9, 72.5); (61.0, 65.8)]. Significant increases in the proportion of providers who counseled PPLW were reported among NP/PAs (25.5 % to 41.6 %) [95 % CIs: (20.1, 30.9); (35.5, 47.7)], and family medicine providers from 28.0 % to 36.5 % [95 % CIs: (24.0, 31.9); (32.4, 40.6)] **(**Table 2**)**. OB/GYNs had the highest proportion of providers having counseled PPLW on emergency preparedness planning at both time points, although they did not show a significant difference over time (49.6 % to 43.3 %) [95 % CIs: (43.4, 55.8); (37.1, 49.4)].

### 3.4 Confidence in counseling on emergency preparedness plans

Compared to 2021, a similar proportion of providers in 2023 reported their confidence counseling pregnant women on emergency preparedness plans as either “Somewhat” or “Very” confident (2021: 53.2 % to 2023: 51.0 %), but there was a significant decline in “Somewhat” confident (38.2 % to 32.8 %)[95 % CIs: (35.7, 40.6); (30.4, 35.2)]. There was a significant increase in the proportion of NP/PAs reporting either “Somewhat” or “Very” confident (43.8 % to 57.6 %) [95 % CIs: (37.7, 50.0); (51.5, 63.7)] with a significant decrease in “Not too Confident” (32.7 % to 19.6 %) [95 % CIs: (26.9, 38.5); (14.7, 24.5)] responses respectively. There was a significant decrease in proportion of OB/GYNs that reported feeling “Somewhat Confident” (49.6 % to 31.7 %) [95 % CIs: (43.4, 55.8); (26.0, 37.5)] and a significantly increased proportion reporting “Not Confident” (5.2 % to 16.3 %) [95 % CIs: (2.5, 8.0); (11.7, 20.8)] (Table 2**)**.

### 3.5 Prevalence of counseling among select characteristics

In the 2023 sample, there was a significant association between age group of provider and having counseled patients in emergency preparedness. Compared to the providers in the youngest age group (<35 years), the providers in older age groups were 48 %–57 % more likely to counsel patients [35–50 years PR: 1.45 (95 % CI: 1.19, 1.88); 51–65 years PR: 1.48 (95 % CI: 1.19, 1.88); >65 years PR: 1.57 (95 % CI: 1.19, 2.08)] (Table 3**)**. Years of practice followed a similar pattern as age. Providers with 11 or more years of practice were 45 %–49 % more likely to counsel when compared to those with 3–5 years of practice [6–10 years of practice PR: 1.26 (95 % CI: 0.98, 1.62); 11–20 years of practice PR: 1.49 (95 % CI: 1.19, 1.89); >20 years of practice PR: 1.45 (95 % CI: 1.17, 2.82)]. Number of patients seen per week by providers showed a significant association with prevalence of counseling. Compared to providers who saw fewer than 100 patients per week, providers who reported seeing ≥100 patients per week were 24 % more likely to have counseled (95 % CI: 1.08, 1.41). No significant differences were observed between provider sex, census regions, community type, practice type or approximate patient household income.

**Table 3:** Emergency preparedness and response counseling practices among healthcare providers by selected characteristics - Fall DocStyles, US, 2023.

	Providers Report Having Counseled		1
	Yes	No	Prevalence Ratio^2^ (95 % CI)
Characteristic	N (%)	N (%)	
Provider Type			
OB/GYN	109 (43.3)	143 (56.7)	1.00
Internist	143 (30.5)	326 (69.5)	0.70 (0.58–0.86)
Family Medicine	194 (36.5)	338 (63.5)	0.84 (0.71–1.01)
NP/PA	104 (41.6)	146 (58.4)	0.96 (0.78–1.18)
Age Group (in years)			
<35	70 (26.0)	199 (74.0)	1.00
35–50	228 (38.6)	362 (61.4)	1.49 (1.19–1.88)
51–65	189 (38.6)	301 (61.4)	1.48 (1.19–1.88)
>65	63 (40.9)	91 (59.1)	1.57 (1.19–2.08)
Years of Practice			
3–5	73 (27.2)	195 (72.8)	1.00
6–10	101 (34.2)	194 (65.8)	1.26 (0.98–1.62)
11–20	155 (40.6)	227 (59.4)	1.49 (1.19–1.89)
>20	221 (39.6)	337 (60.4)	1.45 (1.17–2.82)
Patients Per Week			
<100	252 (32.8)	516 (67.2)	1.00
≥100	298 (40.5)	437 (59.5)	1.24 (1.08–1.41)
Sex^3^			
Male	311 (38.3)	500 (61.7)	1.00
Female	231 (34.2)	445 (65.8)	0.89 (0.78–1.02)
Provider Work Community			
Suburban	281 (35.8)	505 (64.2)	1.00
Urban	204 (37.4)	342 (62.6)	1.04 (0.86–1.24)
Rural	65 (38.0)	106 (62.0)	1.05 (0.87–1.25)
Census region			
Midwest	115 (35.6)	208 (64.4)	1.00
Northeast	117 (36.1)	207 (63.9)	1.01 (0.82–1.25)
South	208 (37.9)	341 (62.1)	1.06 (0.89–1.28)
West	110 (35.8)	197 (64.2)	1.01 (0.82–1.24)

NP – Nurse Practitioner; PA – Physician Assistant; OB/GYN – Obstetrician/Gynecologist

1 EPR3.

2 Unadjusted Prevalence Ratio.

3 16 respondents were excluded from sex (N = 1487) in 2023.

### 3.6 Prevalence of counseling by attitude

Compared to providers who reported emergency preparedness plans are “Not too Important,” providers who reported that emergency preparedness plans are “Somewhat Important” were 3.07 (95 % CI: 1.55, 7.62) times more likely to have counseled patients. Providers who reported plans are “Very Important” were 5.90 (95 % CI: 3.03, 14.50) times more likely to have counseled than those who reported plans are “Not too Important.” These patterns were similar for providers who reported counseling PPLW about emergency preparedness plans is important (Very Important PR: 5.48; 95 % CI: 3.57, 9.17; Ref: “Not too Important”) and provider confidence in counseling on emergency preparedness plans (Very Confident PR: 5.10; 95 % CI: 4.12, 6.43; Ref: “Not too Confident”) (Table 4**)**.

**Table 4:** Emergency preparedness and response counseling practices among healthcare providers by provider attitudes - Fall DocStyles, US, 2023.

	Providers Report Having Counseled^1^		
	Yes	No	Prevalence Ratio^2^
Attitude	N (%)	N (%)	(95 % CI)
*EPR 1: How important are emergency preparedness plans for keeping pregnant, postpartum, or lactating women healthy in disasters or severe weather emergencies?*			
Very Important	417 (49.9)	419 (50.1)	5.90 (3.03–14.50)
Somewhat Important	121 (26.0)	345 (74.0)	3.07 (1.55–7.62)
Not too Important	6 (8.5)	65 (91.5)	1.00
Not Important	1 (4.5)	21 (95.5)	0.54 (0.03–2.93)
*EPR 2: How important is talking with*^3^*pregnant, postpartum, or lactating women about emergency preparedness plans for disasters or severe weather emergencies?*			
Very Important	365 (57.7)	268 (42.3)	5.48 (3.57–9.17)
Somewhat Important	165 (29.2)	400 (70.8)	2.77 (1.78–4.69)
Not too Important	16 (10.5)	136 (89.5)	1.00
Not Important	1 (2.3)	42 (97.7)	0.22 (0.01–1.03)
*EPR 4: How confident are you in talking with*^3^*a pregnant woman about developing an emergency preparedness plan (including preparing an emergency birth kit) in case of a disaster or severe weather emergency?*			
Very Confident	229 (83.6)	45 (16.4)	5.10 (4.12–6.43)
Somewhat Confident	246 (49.9)	247 (50.1)	3.05 (2.43–3.88)
Not too Confident	69 (16.4)	352 (83.6)	1.00
Not Confident	6 (2.4)	247 (97.6)	0.15 (0.06–0.30)

1 EPR3.

2 Unadjusted Prevalence Ratio.

3 ”Talking with” is referred to as “counseling” in the text.

## 4 Discussion

Between 2021 and 2023, we found a significant increase in the proportion of providers reporting emergency preparedness counseling for PPLW as “Very Important”, and a small but significant increase in the proportion of providers having counseled PPLW about developing an emergency preparedness plan, although this remains a minority of providers. Importantly, compared to 2021, there was a significant decrease in OB/GYN's feeling “Somewhat Confident” and a significant increase in those feeling “Not Confident”.

In addition to these decreases in confidence, a non-significant, lower proportion of OB/GYNs reported having provided emergency preparedness counseling. At the same time, significantly larger proportions of family medicine physicians, and NP/Pas had reported counseling. Notably NP/PAs were the only specialty to see an increase in overall confidence while also reporting the largest increase in having counseled PPLW.

These observed increases in counseling may be partially driven by the increase in the proportion of providers reporting emergency preparedness counseling for PPLW as very important. Providers who reported a higher level of importance for having an emergency plan, counseling about emergency preparedness plans or confidence in providing emergency preparedness counseling were more likely to have provided such counseling to PPLW. This association suggests opportunities to increase counseling by raising provider awareness of the importance of emergency preparedness counseling for PPLW, as well as increasing provider confidence through improvement of knowledge and skills for such counseling.

In a previous analysis of 2021 DocStyles data, Meeker described that the most common barriers providers cited for not counseling were not having time (48 %) or lack of knowledge (34 %), and also found that more tenured providers, those with >6 years of experience, were 11–21 % less willing to participate in trainings on emergency preparedness counseling for PPLW (Meeker et al., 2023). Our data from 2023 DocStyles also shows that healthcare providers who were younger (<35 years old) or earlier in their career (3–5 years of practice) were less likely to counsel PPLW on emergency preparedness. A possible cause for the lower counseling rate among these groups is a lack of emergency preparedness training in medical schools, as one study found only 7 of the 156 US allopathic medical schools in 2021 incorporated any disaster response training into their curriculum (Lin et al., 2022). This lack of training raises the possibility that newer healthcare providers have little to no experience with emergency preparedness counseling when starting their professional careers. In comparison, more tenured healthcare providers have possibly gained this experience from post-graduate training or patient situations requiring this type of counseling, although research is needed to identify the causes of this gap between newer and experienced providers. Recent research has continued to identify needs among providers for emergency preparedness training (Meeker et al., 2023; Perihan et al., 2024; Lamberti-Castronuovo et al., 2022) and the efficacy of training (Sreeram et al., 2024; Chia-Huei et al., 2024). This suggests an opportunity for focused interventions, both during professional training (e.g. medical or nursing schools and residency programs) and as continuing education to improve awareness, skills, and confidence surrounding emergency preparedness.

Additionally, providers seeing fewer than 100 patients per week were less likely to have counseled patients than those seeing greater than 100 patients per week. More information is needed to understand the way emergency preparedness counseling is provided in these settings – whether delivered by other personnel (e.g., nurses, front desk staff, social workers) and how information is delivered (e.g., 1-on-1 counseling, group counseling, provision of materials, waiting room videos).

Notably, while overall OB/GYNs remained the most likely group to counsel PPLW (compared to other provider types), they were the only provider type with a decrease (−6.3 percentage points, although not statistically significant) in proportion of providers reporting to have ever counseled patients on developing an emergency preparedness plan. Additionally, OB/GYNs had the largest decrease (−17.2 percentage points) in proportion of respondents reporting feeling “Very” or “Somewhat” confident in counseling on emergency preparedness plans. This change is unlikely to be solely explained by the demographic differences we observed among OB/GYNs between the 2021 and 2023 samples; however we cannot rule out causes due to demographic differences we did not measure. More research is needed to determine reasons for these decreases among OB/GYNs.

Recent studies (2022 & 2023) have shown only about 60 % of women with a recent live birth had developed an emergency preparedness plan (Miller et al., 2023; Strid et al., 2022), highlighting a gap providers can fill by counseling PPLW on developing emergency plans. While emergency preparedness tools and materials exist for PPLW (https://www.cdc.gov/reproductive-health/php/emergency-tools) before, during, and after emergencies, these resources are primarily directed towards public health practitioners and are not tailored to clinicians or healthcare providers. Tailoring materials for a clinical audience may be a strategy to improve delivery of patient safety messages (Noar et al., 2009).

We previously reported on the limitations of DocStyles in Meeker et al. (Meeker et al., 2023), and the following limitations remain applicable to this analysis. Sampling methodology for DocStyles is not random or nationally representative, limiting generalizability to the entire U.S. provider population. However, this survey gathers responses from a large sample of healthcare providers, inclusive of various medical specialties, years in practice, practice setting, and different U.S. census regions. Data in DocStyles are self-reported, and may not be reflective of actual provider practices due to recall and social-desirability bias.

There are additional limitations introduced by this analysis. First, we were unable to determine if there were any providers who were sampled both in 2021 and 2023. However, the surveys in 2021 and 2023 were conducted by different research vendors (SERMO in 2021 and M3 Global Research in 2023) sampling from different pools of providers, decreasing the likelihood of repeated providers. Second, the wording of two questions changed from asking about “women of reproductive age (including pregnant, postpartum, or lactating women)” in 2021 to asking about “pregnant, postpartum, or lactating women” removing the term “women of reproductive age”. While similar, providers may have interpreted these terms differently, particularly the removal of the term “women of reproductive age”.

## 5 Conclusion

These findings show that, while providers are increasingly finding emergency preparedness counseling for PPLW to be important, provider confidence in their ability to provide counseling for pregnant women appears stagnant or decreasing. Notably, fewer OB/GYNs reported feeling confident in providing emergency preparedness counseling – the only provider type with such a decrease. While there was an overall increase in providers reporting to have counseled PPLW, it remains a minority (36.6 %), with less experienced providers and those seeing fewer patients per week less likely to have provided counseling than their counterparts.

With increasing risks to PPLW from disasters, supporting providers with additional resources and training interventions may increase emergency preparedness counseling for PPLW, and ultimately reduce adverse outcomes during and after emergencies. Additional research is needed to identify causes of the stagnation or decrease in counseling among providers, as well as effective emergency preparedness training methodologies for providers and patients.

## Financial disclosures

No financial disclosures were reported by the authors of this paper.

## CRediT authorship contribution statement

**Jerome S. Leonard:** Writing – original draft, Methodology, Formal analysis, Data curation, Conceptualization. **Romeo R. Galang:** Writing – review & editing, Supervision, Methodology, Data curation, Conceptualization. **Grayson Waits:** Writing – review & editing, Validation. **Rebecca Hall:** Writing – review & editing, Conceptualization. **Carrie K. Shapiro-Mendoza:** Writing – review & editing, Conceptualization. **Jessica R. Meeker:** Writing – review & editing, Supervision, Methodology, Formal analysis, Data curation, Conceptualization.

## Disclaimer

The findings and conclusions in this report are those of the authors and do not necessarily represent the official position of the Centers for Disease Control and Prevention.

## Declaration of competing interest

The authors declare that they have no known competing financial interests or personal relationships that could have appeared to influence the work reported in this paper.

## Figures and Tables

**Table:** 

1	Very important
2	Somewhat important
3	Not too important
4	Not at all important
5	Don't know

**Table:** 

1	Very important
2	Somewhat important
3	Not too important
4	Not at all important
5	Don't know

**Table:** 

1	Yes
2	No

**Table:** 

1	Very confident
2	Somewhat confident
3	Not too confident
4	Not at all confident
5	Don't know

**Table:** 

1	Very important
2	Somewhat important
3	Not very important
4	Not at all important
5	Don't know

**Table:** 

1	Very important
2	Somewhat important
3	Not very important
4	Not at all important
5	Don't know

**Table:** 

1	Yes
2	No

**Table:** 

1	Very confident
2	Somewhat confident
3	Not very confident
4	Not at all confident

## Data Availability

Data will be made available on request.

## Appendix A Appendix

2023 Questions:

**EPR1**

In general, how important are emergency preparedness plans for keeping pregnant, postpartum, or lactating women healthy in disasters or severe weather emergencies?

*Select one.*

**EPR2**

How important is talking with pregnant, postpartum, or lactating women about emergency preparedness plans for disasters or severe weather emergencies?

*Select one.*

**EPR3**

Have you ever talked with a pregnant, postpartum, or lactating woman about developing an emergency preparedness plan in case of a disaster or severe weather emergency?

*Select one.*

**EPR4**

How confident are you in talking with a pregnant woman about developing an emergency preparedness plan (including preparing an emergency birth kit) in case of a disaster or severe weather emergency?

*Select one.*

**2021 Questions:**

**EPR1**

In general, how important do you think emergency preparedness plans are for keeping women of reproductive age (including pregnant, postpartum, or lactating women) healthy in disasters (natural or man-made) and severe weather emergencies?

*Select one.*

**EPR2**

How important would you rank talking with women of reproductive age (including pregnant, postpartum, or lactating women) about emergency preparedness plans?

*Select one.*

**EPR3**

Have you **ever** talked with a pregnant, postpartum, or lactating woman about developing an emergency preparedness plan in case of a disaster or severe weather emergency?

*Select one.*

**EPR4**

How confident are you in talking with a pregnant woman about developing an emergency preparedness plan (including preparing an emergency birth kit) in case of a disaster or severe weather emergency?

*Select one.*
